# Next-Generation Sequencing and Triple-Negative Breast Cancer: Insights and Applications

**DOI:** 10.3390/ijms24119688

**Published:** 2023-06-02

**Authors:** Domenico Tierno, Gabriele Grassi, Serena Scomersi, Marina Bortul, Daniele Generali, Fabrizio Zanconati, Bruna Scaggiante

**Affiliations:** 1 Department of Life Sciences, University of Trieste, 34127 Trieste, Italy; domenico.tierno@phd.units.it (D.T.); ggrassi@units.it (G.G.); 2 Breast Unit-Azienda Sanitaria Universitaria Integrata Giuliano Isontina ASUGI, University of Trieste, 34149 Trieste, Italy; serena.scomersi@asugi.sanita.fvg.it; 3 Department of Medical, Surgical and Health Sciences, University of Trieste, 34149 Trieste, Italy; m.bortul@fmc.units.it (M.B.); dgenerali@units.it (D.G.); fabrizio.zanconati@asugi.sanita.fg.it (F.Z.); 4 Azienda Socio-Sanitaria Territoriale di Cremona-ASST, Breast Cancer Unit and Translational Research Unit, 26100 Cremona, Italy

**Keywords:** 454-pyrosequencing, Illumina, Ion Torrent, next-generation sequencing, triple-negative breast cancer

## Abstract

The poor survival of triple-negative breast cancer (TNBC) is due to its aggressive behavior, large heterogeneity, and high risk of recurrence. A comprehensive molecular investigation of this type of breast cancer using high-throughput next-generation sequencing (NGS) methods may help to elucidate its potential progression and discover biomarkers related to patient survival. In this review, the NGS applications in TNBC research are described. Many NGS studies point to *TP53* mutations, immunocheckpoint response genes, and aberrations in the PIK3CA and DNA repair pathways as recurrent pathogenic alterations in TNBC. Beyond their diagnostic and predictive/prognostic value, these findings suggest potential personalized treatments in PD -L1-positive TNBC or in TNBC with a homologous recombination deficit. Moreover, the comprehensive sequencing of large genomes with NGS has enabled the identification of novel markers with clinical value in TNBC, such as *AURKA*, *MYC*, and *JARID2* mutations. In addition, NGS investigations to explore ethnicity-specific alterations have pointed to *EZH2* overexpression, *BRCA1* alterations, and a *BRCA2*-delaAAGA mutation as possible molecular signatures of African and African American TNBC. Finally, the development of long-read sequencing methods and their combination with optimized short-read techniques promise to improve the efficiency of NGS approaches for future massive clinical use.

## 1. Introduction

Triple-negative breast cancer (TNBC) is an aggressive subtype of breast cancer characterized by the absence of estrogen receptor (ER), progesterone receptor (PR), and human epidermal growth factor receptor 2 (HER2) [1,2]. According to the American Cancer Society, TNBC accounted for 10% of all breast cancers diagnosed in the United States in 2019, with a particularly high incidence among young women and women of African or Hispanic descent [3,4]. This tumor has a high risk of recurrence and metastasis, resulting in a 5-year survival rate of approximately 25% when diagnosed at stages III and IV [5,6]. In addition, the term TNBC was coined in the 2000s to refer to breast cancers lacking ER, HER2, and PR, but subsequent analyses have shown substantial molecular, histologic, and clinical differences among TNBC patients. This wide heterogeneity requires the comprehensive molecular profiling of the different TNBC subtypes to stratify its diagnosis and plan an appropriate and specific therapeutic approach [7]. The clinical picture of TNBC is exacerbated by the inadequacy of its conventional treatments: endocrine and anti-HER2 therapies for hormone-receptor-positive and HER2-positive breast cancer are ineffective for TNBC patients due to their absence of hormone and HER2 receptors [8]. This limitation highlights the need to discover new molecular targets to improve our therapeutic options. A molecular characterization of TNBC may provide insight into the intrinsic mechanisms regulating the tumor onset and progression. Several genomic studies have indicated a high mutational burden compared to other breast cancer subtypes and a significant rate of copy number alterations (CNA) [9,10]. Notably, CNA events occur in all genomes, with recurrent 1q, 8q, and 10q gains, *FGFR* and *EGFR* amplifications, and 5q and 8p or *PTEN* losses [11]. *TP53* is the most frequently mutated gene (about 80% of TNBC cases), followed by *PIK3CA* (25–30%), *KMT2C*, *PTEN*, and *RB1* (about 5%) [7,12].

The advances in the molecular profiling of TNBC are diverse, such as classifications into different molecular subtypes. Several efforts agree to distinguish four TNBC groups at the level of genes: basal-like immune activated (BLIA) with *TP53* mutation and a homologous recombination DNA repair deficiency (HRD) [13]; basal-like immune suppressed (BLIS), which is like BLIA, except for those with a few tumor-infiltrating lymphocytes [14]; mesenchymal-like (MES) with a low mutational burden and *PIK3CA* mutation [1]; and luminal androgen receptor (LAR), which is characterized by the oncogenic activation of the ER pathway [15]. The molecular subtyping of TNBC has some important clinical implications: for example, the LAR subtype responds to both anti-estrogen and anti-androgen therapies, despite its ER negativity [16]. In addition, the BLIA and BLIS subtypes respond very well to PARP inhibitors (PARPi) due to their HRD. Indeed, these tumors are highly dependent on efficient, single-strand repair machinery (mediated by PARP). The inhibition of PARP therefore leads to an accumulation of DNA single-strand damage, which becomes irreparable double-strand lesions [17]. The OlympiAD clinical trial demonstrated that TNBC patients with *BRCA* germline mutations showed a significant improvement in progression-free survival after treatment with the PARPi olaparib in comparison to the standard chemotherapy [18,19].

Beyond molecular classification, comprehensive knowledge of TNBC’s mutational signatures paves the way for specific and efficient therapeutic approaches. For example, PD-L1 (cell death protein ligand 1) has been proposed as a novel therapeutic target for TNBC. The latter is overexpressed in several tumors because it can bind its ligand PD-1 on the surface of T cells, inducing the inhibition of the immune response [20]. Therefore, TNBC patients with a high PD-L1 expression may overcome the immune response, resulting in poor outcomes [21]. Several clinical trials have tested the clinical efficacy of PD-L1 inhibitors such as atezolizumab and pembrolizumab in TNBC patients with a high PD-L1 expression. The promising results of these clinical trials led to atezolizumab being approved by the FDA for the treatment of metastatic TNBC in 2019 [22,23]. However, not all TNBC patients with PD-L1 expression can benefit from treatment with PD-L1-targeting drugs [24,25]. Therefore, an investigation of the genomic landscapes of these patients may be helpful for the prediction of treatment with immune-targeting drugs. Another potential target for the treatment of this tumor is the PIK3CA/AKT pathway, which is dysregulated in approximately 25–30% of TNBC patients [26]. Several clinical trials have investigated the clinical relevance of AKT inhibitors in the treatment of TNBC [27]. One example is the LOTUS trial, which showed that patients with *PIK3CA*/*AKT* mutations had a more significant improvement in progression-free survival after treatment with the AKT inhibitor Ipatasertib than patients without mutations [28].

As described so far, a comprehensive understanding of the molecular pathways that underpin TNBC progression can improve our clinical options against this tumor. Therefore, the development of rapid, cost-effective, and efficient technologies for the molecular profiling of TNBC patients is of great importance.

NGS technologies are constantly improving to develop faster, cheaper, and more accurate methods for sequencing large genomic samples. In contrast to first-generation techniques, the high throughput of these methods has dramatically increased the output of their sequencing data through comprehensive analyses of broad genomic regions. These innovations have led to a deeper knowledge of the genome and molecular biology, especially with respect to human diseases and their clinical treatment. This review aims to summarize some relevant results of the application of NGS techniques in Triple-Negative Breast Cancer research. The articles reviewed were searched in PubMed using the keywords “NGS” AND “TNBC”, with the start year for the search being 2014 and only original articles in English being considered. All closely relevant articles dealing with NGS in TNBC patients were selected. Studies in vitro were not included, as our goal was to emphasize the clinical aspect of NGS. Studies were categorized into the molecular characterization of TNBC, novel diagnostic biomarkers for TNBC, and novel potential therapeutic targets. In addition, the studies reviewed were selected to provide an overview of the most commonly used NGS methods in investigations of TNBC. Representative NGS studies on liquid biopsies were highlighted to demonstrate the versatility of these innovative sequencing methods. Studies on ethnicity-specific molecular signatures were selected for African populations, as TNBC is particularly prevalent in these women. Finally, initial studies on the latest NGS techniques were mentioned to demonstrate the continued development of these methods for clinical research.

## 2. Next-Generation Sequencing

The history of DNA and RNA sequencing methods has undergone a remarkable evolution in the last 15 years with the development of next-generation sequencing (NGS) techniques. These high-throughput methods have improved our knowledge of molecular biology by sequencing large genomes on a large scale [29]. Although the term “next generation” suggests a homogeneous group of new generation methods, NGS techniques are characterized by continuous improvements and advancements. Accordingly, the classification of sequencing methods into only two groups, “next-gen” and “old-gen”, can be confusing. Today, DNA/RNA sequencing methods are classified into four different generations [30]. A brief description is given below.

### 2.1. First-Generation Sequencing

First-generation sequencing methods include the basic techniques that preceded the introduction of NGS. The most common methods are Sanger sequencing via synthesis (SBS) and Maxam–Gilbert chemical cleavage (MGC).

MGC is based on the specific chemical modification of DNA and the subsequent cleavage of the DNA backbone at the modified nucleotides. The obtained fragments are then separated in a gel according to their size to obtain a ladder of DNA fragments with a known nucleotide end [31].

In SBS, DNA or RNA sequencing is achieved by chain-terminating dideoxy nucleotides (ddNTP). The absence of 3′- OH interrupts the DNA chain during polymerization; therefore, phosphodiester bonds cannot be formed. Each ddNTP is labeled with a specific fluorophore to allow for the detection of DNA fragments of different sizes using capillary electrophoresis [32]. Despite the efficiency of NGS techniques, SBS is still widely used for applications where a high throughput is not required. Its most common applications range from the validation of plasmid constructs or PCR products to testing enzyme action on DNA molecules [33,34].

### 2.2. Second-Generation Sequencing

The first properly named NGS techniques belong to this group. The development of these sequencing methods triggered the need for sequencing large genomes with a higher and cheaper throughput [30]. Depending on the working principle, two categories of second-generation sequencing methods can be distinguished:

Next generation sequencing via hybridization (NGSH): this is based on a microarray of oligonucleotides with known sequences and positions and their hybridization with a fluorophore-labeled target DNA [35]. Sequencing via hybridization is commonly proposed in routine clinical practice for the identification of disease-related SNPs (single-nucleotide polymorphisms) or chromosomal abnormalities (such as deletions and amplifications) [36].

Next-generation sequencing via synthesis (NGSS): this represents the evolution of Sanger sequencing via synthesis. These methods can only be applied to short DNA fragments (usually 300–500 nucleotides) and are characterized by a considerable error rate, but their high data throughput allows for comprehensive genome coverage and avoids the effects of the error rate [37].

**Figure 1:**
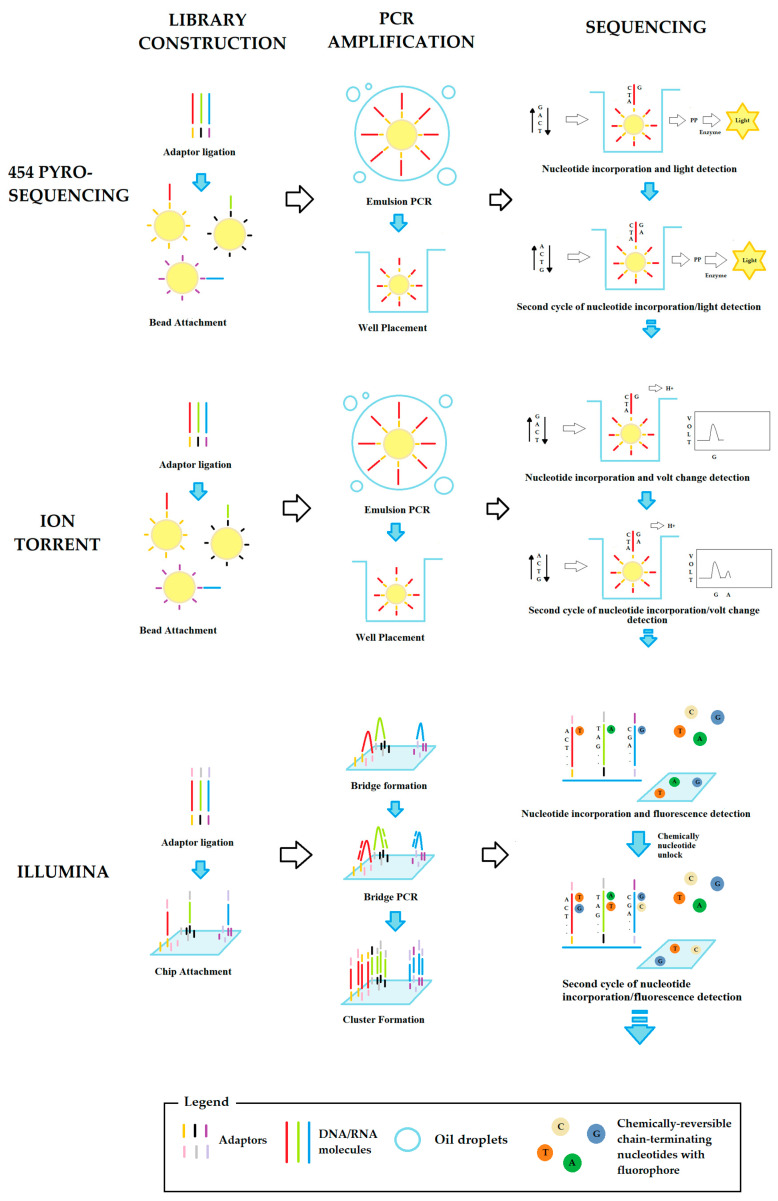
Schematic workflow of second-generation sequencing approaches.

NGSS techniques are based on different sequencing approaches, usually based on the individual isolation of DNA molecules in millions of chambers, wells, or specific spots on a chip. Among the many systems, the most common (schematically shown in Figure 1) are:

454 Pyrosequencing: in this approach, each DNA fragment of 600–800 nucleotides is conjugated with an oligonucleotide adaptor complementary to a DNA sequence on a bead. Ideally, each bead can bind a single DNA fragment using specific adaptors. The DNA fragments on the beads are then amplified with an emulsion PCR (emPCR) and each bead is transferred to a picoliter-sized chamber. Each chamber is flooded alternately with one of the four nucleotides. When the correct nucleotide is incorporated, a pyrophosphate molecule is released and recorded using a light-generating reaction. Although this approach is rarely used, it represents an efficient choice for whole genome sequencing and metagenome analyses, due to the length of the analyzed DNA fragments [38].

Ion Torrent: this approach is quite similar to 454 pyrosequencing but differs in the method used to detect the correct insertion of nucleotides. When a nucleotide is inserted into a growing chain, it releases a hydrogen ion that changes the pH of the solution, which can be recorded as a voltage change by an ion sensor. This detection system is faster than that of 454 pyrosequencing because it does not require a camera or light source [39]. For this reason, the ion torrent method is widely used for various applications, such as de novo sequencing or DNA alteration detection.

Illumina technology: this is based on an innovative amplification method called “bridge PCR”. In this method, each DNA fragment of about 500 nucleotides is functionalized at both ends with two oligonucleotide adaptors complementary to several DNA sequences on a solid support. In this way, the DNA fragment is anchored to the solid support by the two adaptors like a bridge and can serve as a template for amplification in clusters of about 1000 replicates. The clusters are sequenced by chemically reversible chain-terminating nucleotides labeled with a fluorophore specific for each of the four nucleotides. The insertion of a modified nucleotide results in the temporary blockage of the growing chain, and the specific fluorescence is recorded. After the detection, the modified nucleotide is chemically unlocked and a new cycle of insertion/detection can begin [37]. Illumina technology is by far the most widely used NGS technique and supports a variety of applications, including DNA or RNA sequencing, metagenomics, CHIP -seq, and methylome analyses [40].

### 2.3. Third-Generation Sequencing

**Figure 2:**
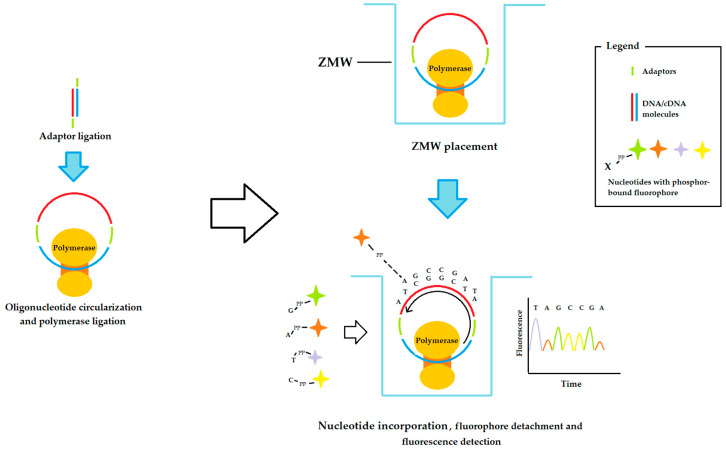
Schematic workflow of Pacbio sequencing approaches. ZMW: zero-mode wave (1 zeptoliter-sized well).

In this generation, innovations in NGS approaches aim to increase the length of the DNA or RNA fragments analyzed. The leading method in this case is Pacbio sequencing, also called single-molecule real-time sequencing (SMRT), an innovative system that can sequence very long fragments (30–50 kilobases). Its workflow begins by functionalizing the DNA or RNA fragments to be sequenced with special adaptors that allow the molecule to circulate. The resulting circular DNA/RNA is then bound to a special polymerase and the complex is placed on the bottom of a zero-mode waveguide (ZMW) that is the size of a zeptoliter. The small size of the ZMW (1 zeptoliter = 1 × 10–21 L) directs the incident light to a small area so that only the bottom of the well can be illuminated and imaged. Each well is then flooded with a mixture of the four nucleotides labeled with a phosphor-bound fluorophore. In this way, the incorporation of a nucleotide into the growing DNA chain detaches the fluorophore, which then floats away from the illuminated region. Imaging synchronized with the nucleotide insertion rate thus allows for the detection of light from the fluorophore only at the time of insertion. In addition, this detection approach can provide information on the presence of various base modifications, such as adenine and cytosine methylation, as the latter alters the nucleotide incorporation rate [41]. A scheme of this sequencing method is illustrated in Figure 2.

It is essential to emphasize that SMRT sequencing is associated with a considerable error rate. However, its high number of parallel sequencing runs due to the small size of the ZMW can overcome this problem. The applications of SMRT technology are numerous, especially for the detection of base changes, and it is often used in combination with other NGS methods to increase the sequencing accuracy [42]. The continuous optimization of the SMRT workflow makes this NGS technique a good choice for the analysis of large genomic samples.

### 2.4. Fourth-Generation Sequencing

Like the third generation, the sequencing methods of the fourth generation are focused on increasing the analyzed sample length. The fundamental principle of these sequencing approaches is the passage of the DNA or RNA molecule through a hole coupled with a detector system. Theoretically, around 1000 kb can pass through a single hole, which leads to a consistent sequencing throughput [43]. According to the materials used, it is possible to distinguish two types of nanopore systems:

Solid-state sensor technology: this is based on metal or metal alloy chips with nanometer-sized pores, through which the DNA/RNA molecules pass [44].

**Figure 3:**
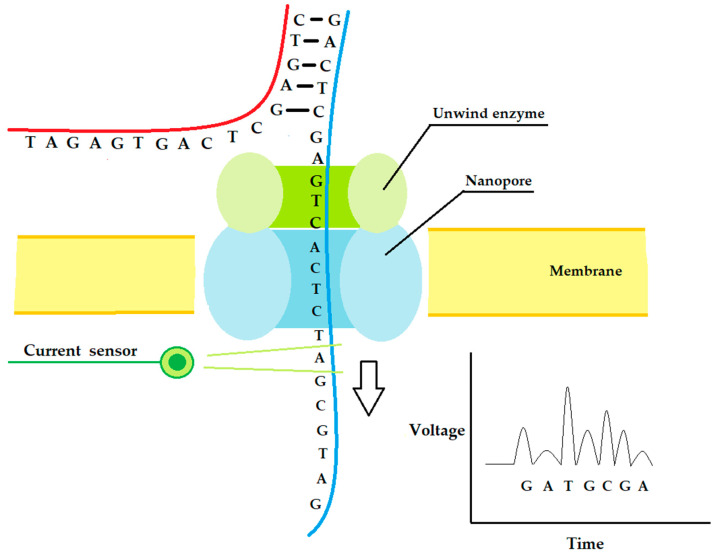
Scheme of working principle of nanopore sequencing approaches.

Biological membrane systems: this approach uses an electrically resistant polymer membrane with nanopores generated by transmembrane proteins, usually the alpha-hemolysin or the Mycobacterium smegmatisporin A (MspA). The long double-strand DNA/cDNA molecule is initially coupled with an enzyme complex made of a highly processive DNA polymerase (phi29), a DNA helicase, and an exonuclease I, which is able to unwind and ratchet the DNA molecule through the pore at a constant rate. As a nucleotide passes through the pore, it generates an electronic signal characteristic of each of the four nucleotides. In this way, it is possible to sequence the analyzed DNA/cDNA sample knowing the oligonucleotide pass rate through the pore. The leader in biological membrane systems is the MinION platform commercialized by Oxford Nanopore Technologies (ONT), with a small handle size valuable device for its portability and low space requirement [45]. A scheme of this sequencing method is illustrated in Figure 3.

Like other NGS technologies, fourth-generation sequencing methods are characterized by a high error rate, which is circumvented by their high numbers of parallel reads. Nanopore systems are typically used for sequencing environmental and metagenomic samples; in space stations, for example, they are used to identify bacterial strains. Like SMRT sequencing, these methods can identify base changes by altering the standard electrical signals of the nucleotides passing through the nanopore [46].

In summary, NGS techniques are a useful tool for deepening our knowledge of genomics. The large pool of sequencing data generated by NGS has contributed to the discovery of new characteristic, diagnostic, and prognostic biomarkers for a variety of diseases and has improved our therapeutic options. Nevertheless, NGS techniques are characterized by a higher error rate and shorter analyzed DNA/RNA fragments than first-generation techniques, which retain their clinical utility, particularly in detecting known molecular aberrations. In addition, the results of NGS methods are more expensive than those of the first generation, which hinders their massive use. However, the advances in NGS techniques in terms of faster, more accurate, and less expensive sequencing, combined with their high throughput, are enabling their wider incorporation into routine clinical practice.

## 3. NGS Technologies in TNBC Research

TNBC is a rare form of breast cancer with aggressive behavior and a high risk of recurrence, resulting in generally poor outcomes [3]. In this perspective, the optimized sequencing technologies of the second generation can help to discover new diagnostic, predictive, and prognostic biomarkers for improving patient survival rates.

TNBC is characterized by a high molecular, histological, and clinical heterogeneity [6]. Several efforts have focused on the identification of biomarkers using NGS techniques for TNBC diagnosis stratification.

### 3.1. NGS Analysis of Markers for TNBC

Twelve studies have analyzed TNBC markers using NGS, as shown in Table 1, most of which used formalin-fixed, paraffin-embedded samples. Four studies were performed in a liquid biopsy using peripheral blood.

Liang et al. investigated the mutation status of 91 breast cancer-related genes in 156 inflammatory breast cancer (IBC) patients (among which 51 were TNBC cases) using the Illumina platform. The sequencing results from the fresh or frozen biopsies were compared to a control group of 191 non-IBC primary breast cancer patients from the TGCA database. The Illumina analysis showed a higher somatic mutation frequency for *TP53, NOTCH1/2, MYH9, BRCA1/2, ERBB4, POLE, FGFR3, ROS1, NOTCH4, LAMA2, EGFR, ESR1, THBS1*, and *CASp8*, and a lower somatic mutation frequency for CDH1 in the IBC patients in comparison to the non-IBC ones. Upon grouping these genes in cellular pathways, it was possible to observe how the DNA repair, NOTCH, and RAS pathways were more altered in the IBC group than the non-IBC group, paving the way for specific IBC therapies. Moreover, the PIK3CA/AKT pathway was often altered in IBC (especially in TNBC subtypes) and was associated with a poor metastasis-free survival [47].

An exciting contribution to TNBC molecular profiling was provided by Srour et al., who compared the expression levels of 2567 cancer-related genes in 14 pairs of ER+ primary sites and paired axillary lymph node metastasis (ALN), and in 17 pairs of TNBC primary sites and paired ALN metastasis. In both subgroups, an Illumina analysis revealed multiple genes with different expression levels between the primary and metastatic sites. A comparison between the TNBC and ER+ results showed that 97 common upregulated genes and 115 common downregulated genes were found in the ALN metastasis in comparison to the primary sites [48]. Another experiment by Srour et al. on the same patient pool showed an overexpression of anti-apoptosis genes (*BIRC3, TCL1A, FLT3, and VCAM1*) and a downregulation of the genes regulating the microenvironment (*MMP2, MMP 3, MMP 7, MMP 11, MMP14, COL1A1, COL1A2, COL3A1, COL5A1, COL5A2, COL6A6, COL11A1*, and *COL17A1*) in ALN metastasis compared to the TNBC primary sites. Despite the small patient cohort, these results suggested a change in the transcriptome of TNBC-invasive cells that increases their metastatic potential [49].

Dillon et al. examined the mutation profiles of 20 TNBC patients using an NGS assay called JAX-CTP. This assay was based on a clinically validated panel of SNPs, copy number variations, insertions, and deletions commonly detected in 358 cancer-related genes. The Illumina sequencing of formalin-fixed, paraffin-embedded (FFPE) biopsies revealed *MYC* amplification in 75% of the patients examined, while *TP53, AURKA*, and *KDR* mutations were present in 6 of 20 cases (30%) [50]. *MYC* is a transcription factor that regulates approximately 15% of all human genes, including the genes related to cell proliferation and survival. *MYC* dysregulation is indeed involved in tumorigenesis in various tissues, inducing epithelial–mesenchymal transition (EMT), angiogenesis, and tumor cell immortalization in the BLIS and BLIA subsets of TNBC [51]. These results suggested that *MYC* amplification is a molecular signature for basal-like TNBCs, as has been previously reported in the literature [52]. The high mutational frequency of *AURKA* in the studied cohort of patients was very interesting, as this plays a role in promoting *MYC* expression in breast cancer stem cells [53]. The AURKA inhibitors alisertib and TAS -119 have provided encouraging results in ongoing studies [54,55], suggesting *AURKA* as an emerging target for the treatment of TNBC. However, the small patient population analyzed by Dillon et al. needs further investigation to confirm these results.

Instead, Li et al. focused on the different histologic groups of TNBC, particularly invasive ductal carcinoma, without special type (NST) and special type (ST). The NST patients were distinguished from those with ST by the absence of special histological features found in a microscopic examination, which classify them into the typical ST subtypes such as medullary, metaplastic, and apocrine carcinomas [56]. They performed an NGS survey using the Illumina platform on the plasma of 89 TNBC patients (72 NSTs and 17 STs) to investigate the mutation statuses of 520 cancer-related genes. The sequencing results showed a different mutation frequency between the two histological subtypes: in NST, the most frequently mutated genes were *TP53* (88.7%), *PIK3CA* (26.8%), and *MYC* (18.3%), whereas in ST, they were *TP53* (68.8%), *PIK3CA* (50%), *JAK3* (18.8%), and *KMT2C* (18.8%). Although *TP53* and *PIK3CA* are the most frequently mutated genes in both subgroups, significantly lower *TP53* and higher *PIK3CA* mutation rates are observed in ST in comparison to NST. This finding provided genetic evidence for the partially different molecular mechanisms underlying the tumor growth in these two TNBC subgroups. Moreover, the efficacy of drugs targeting the PIK3CA pathway may be higher for STs than for NSTs [57].

Beyond diagnostic stratification, the molecular profiling of TNBC with NGS techniques can provide novel biomarkers for investigating the clinical value of genomic alterations. An optimal example is provided by Pop et al., who analyzed the most frequent alterations of 46 genes that play well-defined roles in cancer and their clinical significance in a retrospective study of 96 TNBC patients. Pop et al. performed Ion Torrent sequencing on 30 FFPE tissues from the TNBC cohort and validated the results in all 96 cases. Consistent with other previously described studies, the analysis showed a high mutation frequency for *TP53, KDR, PIK3CA, ATM, AKT1, KIT, ERBB4, FGFR3*, and *MET*. In particular, the presence of *AKT1* rs3730358, *KDR* rs34231037 (c.1444T > C), *KIT* rs3822214 (c.1621A > C), *TP53* rs28934576 (c.818G > A), and *BRCA1/2* class 5 mutations was associated with a worse survival in the patients studied [58]. Of note, these results demonstrated the importance of discovering novel mutations in TNBC for the development of personalized therapeutic treatments.

Its high risk of recurrence is one of the main problems in the clinical management of TNBC. From this point of view, Balko et al. investigated the molecular footprint that determines the chemoresistant response of TNBC tumor cells. Accordingly, they performed a mutational analysis of 182 cancer-related genes in 85 TNBC patients after NAC and in 20 comparable biopsies before treatment with the Illumina platform. The study showed a high mutation frequency in several genes useful for targeted therapies in relapsed cases after NAC: *PTEN* (PI3K and AKT inhibitors), *JAK2* (ruxolitinib or tofacitinib), *CDK6*, *CCND1*, *CCND2*, *CCND3* (CDK4/6 inhibitors), and *IGF1R* (dalotuzumab) [59]. This study underlined the importance of routine molecular analyses in follow-ups of TNBC patients in order to improve their outcomes with the appropriate adjuvant drugs. From this point of view, NGS can be a valuable choice, as it is compatible with clinical timing.

Similar to Balko et al., another group attempted to delve into the molecular signature of treatment-resistant TNBC. Lips et al. performed the molecular profiling of 56 TNBC biopsies before NAC using SOLiD 5500xl, an NGS platform based on library amplification using emulsion PCR and sequencing with fluorescent barcodes. The latter are libraries of specific oligonucleotides functionalized with different fluorophores. When an oligo anneals to its complementary sequence on the analyzed DNA/RNA, the fluorophore is enzymatically cleaved and a camera records the colored light emission. The image data are then converted into spatial data using software to sequence the analyzed molecule [60]. In this study, the SOLiD system was used to analyze the pathogenic variants of 1977 tumorigenesis-related genes in the patient cohort and their correlation with the treatment responses. For each patient, normal DNA was extracted and sequenced from their plasma samples. Unfortunately, the analysis revealed no significant difference in the mutation rates between the patients who responded to treatment and those who were resistant. However, *PIK3CA* mutations were observed exclusively in patients with the *BRCA1* wild-type [61]. This information may provide new predictive biomarkers for the efficacy of PARPi and drugs targeting the PIK3CA pathway.

Xiangmei et al. focused on the molecular characterization of TNBC patients who had not achieved a pathological complete response (non-pCR) after NAC. Here, the 14 post-NAC TNBC patients analyzed were divided into 7 cases with a short disease-free survival (DFS, within 12 months) and 7 others with a DFS of more than one year. Illumina sequencing was used to examine 422 cancer-related genes in the DNA of FFPE, while matched plasma samples were sequenced to detect germline mutations. The analysis revealed a higher mean number of mutations in the short DFS group than in the long DFS group (6 vs. 4.3), indicating tumor mutation burden (TMB) as a possible marker of recurrence. Moreover, mutations of *PTPN13* and *JARID2* were found only in patients from the short DFS group. A parallel *JARID2* knockout experiment at MDA-MBA -231 (a TNBC cell line) showed a decrease in E-cadherin and an increase in vimentin, *MMP7*, and *MMP9* expression, suggesting a possible role of *JARID2* in epithelial–mesenchymal transition (EMT) [62]. These results highlight the prognostic value of TMB and *JARID2* and represent a new potential therapeutic target for TNBC. The plasma analysis showed no returned germline mutations among the 72 pathogenic mutations found in both groups. Like Lips et al. [61], in this study, the DNA from the plasma was sequenced using NGS techniques and used as a control group. Liquid biopsies represent a useful clinical tool due to their non-invasive collection, contrary to standard solid biopsies. The faster sampling of peripheral blood and the high throughput of NGS techniques allow for a solid control data group, resulting in a more accurate analysis in a non-invasive way. However, plasma can also be used as a principal tumor DNA source, as Li et al. showed [57]. These three studies highlight the potentiality of liquid biopsies for tumor research with NGS techniques.

Heeke et al. contributed to TNBC molecular profiling for predicting responses to treatment. Their study was performed on 4647 breast cancer cases (including 1568 TNBCs) using the NGS-592 Sure-Selected XT, an Illumina-based FFPE-specific assay targeting a pan-carcinoma of 592 genes. Specifically, the mutation statuses of DNA-repair-associated genes (*ARID1A, ATM, ATRX, BAP1, BARD1, BLM, BRCA1/2, BRIP1, CHEK1/2, FANCA/C/D2/E/F/G/L, KMT2D, MRE11, NBN, PALB2, RAD50/51/51B*, and *WRN*) and chemoresistant-related genes (*AKT1, AR, ARID2, ATR, AURKA/B, BCL7A, BCL11A/B, BRAF, CDK4/6, CDKN2A, EGFR, ERBB2, ERBB3, ERBB4, ESR1, IDH1, IDH2, JAK1, JAK2, KIT, MET, MTOR, NTRK1/2/3, PBRM1, PD -L1, PIK3CA, POLE, RB1, RET, SMARCB1, SMARCE1, SMARCA4, SMO,* and *SS18L1*) were performed. The analysis revealed that 17.9% of all cases had at least one mutation in the genes involved in the recombination mechanisms of DNA repair homologs. These mutations were found in 18.2% of the TNBC cases [63]. Moreover, breast cancers with homologs recombination deficiencies (HRD) are characterized by a higher tumor mutational burden, PD-L1 expression, and PIK3CA pathway alteration rate in comparison to ones without HRD. The frequent co-occurrence of DNA repair dysfunctions with other response markers to immunotherapy suggests a new possible combinatorial therapeutic approach for TNBCs with HRD.

Immune checkpoint inhibitors (ICPIs) represent a group of drugs that are increasingly used in the treatment of malignancies [64,65]. In TNBC, several clinical trials of PD-L1-targeted drugs have produced positive results, with atezolizumab being approved by the FDA in 2019 [23]. The study by Hoda et al. aimed to investigate the frequency of PD-L1 alterations in TNBC and their associated genomic landscape. To this end, 164 FFPE samples from 158 primary and metastatic TNBC patients were analyzed using MSK-IMPACT, an FDA-approved assay from Illumina that detects 468 major cancer-related genes. Immunohistochemistry (IHC) was used to evaluate PD-L1 positivity. The analysis showed that 47.4% of cases had PD-L1 positivity and that the latter was more common in primary TNBC than metastatic. Interestingly, Hoda et al. reported several benefits of anti-PD-L1 therapy in metastatic patients with PD-L1 negativity. According to the authors, IHC results may be influenced by the different methods of collecting primary and metastatic samples (resection and core needle biopsies, respectively). The NGS analysis revealed no significant difference in the genomic assets between the PD-L1-positive and PD-L1-negative TNBCs, except for *CBFB*. In fact, mutations in this gene are more frequent in patients without PD-L1 expression than in PD-L1-positive ones [66]. It has been reported that *CBFB* mutations usually occur together with mutations in the genes associated with the luminal androgen receptor subset (LAR) of TNBC, such as *AKT1* and *CDH1* [14,67]. Finally, the study indicated that *CBFB* mutations and PD-L1 negativity may represent novel molecular signatures for LAR TNBCs.

Tan et al. [68] investigated the genomic landscape underlying the response to ICPI treatment. To this end, they performed an Illumina assay of 457 cancer-related genes on plasma samples from 11 ICPI-treated TNBC patients. Specifically, they collected and analyzed circulating cell-free tumor DNA (ctDNA) from each patient before the ICPI treatment. Although the number of patients studied was small, this work is interesting because the analysis revealed a shorter progression-free survival after ICPI treatment in patients with deletions of *CYP2D6* and gains in CNV of *NAS, BCL2L1, H3F3C, LAG3, FGF23, CCND2, SESN1, SNHG16, MYC, HLA-E,* and *MCL-1*. The study suggested these 12 genes as novel predictive biomarkers of ICPI treatment efficacy in TNBC. Notably, among these genes, *GNAS, BCL2L1, LAG3, CCND2, SNHG16, MYC, HLA-E*, and *MCL-1* were involved in cell progression, metastasis, apoptosis inhibition, and immune evasion in breast cancer [69,70,71,72,73,74,75].

Another contribution to the identification of the genes associated with responses to immunotherapy in TNBC was made by Sivapiragasam et al. They performed the comprehensive genomic profiling of 3831 metastatic breast cancers (1237 ER+, 1953 HER2+, and 641 TNBC) using FoundationOne CDx, an Illumina-based assay, to detect genetic alterations in 324 genes, as well as genomic signatures such as microsatellite instability (MSI) and tumor mutation burden (TMB). The analysis showed that 42.6% of the ER + cases, 12.1% of the HER2+ cases, and 56.4% of the TNBC cases had genomic alterations associated with a response to ICPIs. The coupled IHC assay indicated PD-L1 positivity in 13%, 33%, and 47% of the ER+, HER2+, and TNBC cases, respectively, confirming the literature data on the high frequency of PD-L1 expression in the triple-negative subtype [76,77]. An NGS validation of the PD-L1 status revealed a low CNA rate (1–2% of all cases), ruling out the amplification of this gene as the main cause of its high expression in metastatic breast cancer [78]. An overall analysis of the breast cancer tissues showed genomic alterations in *STK11* in 2% of the TNBC cases and in *MDM2* in 3% of the TNBC cases. The latter has already been associated with resistance to ICPIs, as it attenuates the immune response [79,80], but few studies have considered it in breast cancer. In conclusion, this trial was useful for demonstrating how comprehensive genomic profiling using NGS can aid with ICPI treatment by identifying biomarkers of resistance, such as *STK11* and *MDM2*.

Tyrosine receptor kinases (TRKs) are an important class of transmembrane receptors involved in cell proliferation and survival pathways. Among them, neurotrophic TRK (*NTRK*) is of interest in tumors because the aberrant fusion of its kinase domains with other genes can lead to a ligand-independent activation of the receptor [81]. In light of this, RTK inhibitors (RTKi) may offer clinical benefits as effective adjuvant drugs in patients with NRTK fusion. Wu et al. conducted a retrospective study to evaluate the NTRK statuses in 305 TNBC patients and to investigate the potential clinical applications of RTKi in TNBC treatment. These *NTRK* statuses were assessed using IHC, FISH, and Illumina NGS sequencing. The IHC and FISH analyses showed that 11.15% of cases had NRTK fusion, but an NGS validation showed no positivity [82]. Although the results precluded the use of RTKi in TNBC, this trial represents an optimal example of the use of NGS for the validation of routine clinical procedures.

**Table 1:** Summary of the experimental methods and main findings from reported next-generation sequencing (NGS) studies in triple-negative breast cancer (TNBC) research. Abbreviations: CNV (copy number variation); DFS (disease-free survival); ER+ (estrogen receptor positive breast cancer); FFPE (formalin-fixed paraffin-embedded); HER2+ (HER2 positive breast cancer); IBC (inflammatory breast cancer); ICPI (immune checkpoint inhibitor); IHC (immunohistochemistry); LAR (luminal androgen receptor); MSI (microsatellite instability); NAC (neo-adjuvant chemotherapy); NR (not reported); NST (no special-type tumor); PFS (progression-free survival); PR+ (progesterone receptor positive breast cancer); ST (special-type tumor); TMB (tumor mutational burden); TNBC (triple-negative breast cancer); and yrs (years).

Patient Population	Median Age (Min-Max)	Source of Sample	Target	NGS Platform	Main Finding	Ref.
156 IBC patients (51 TNBC)	53 yrs (23–84)	Fresh or frozen biopsy	Mutations of 91 breast cancer-related genes	Illumina	DNA repair, NOTCH, RAS and PIK3CA signaling pathways were more frequently altered in IBC patients (especially TNBC) than in non-IBC patients	[47]
14 ER+ patients with paired metastasis/primary samples, 17 TNBC patients with paired metastasis/primary samples	ER+ patients: 61 yrs (35–79); TNBC patients: 62 yrs (30–88)	FFPE	Expression level of 2567 cancer-associated genes	Illumina	97 upregulated genes and 115 downregulated genes in metastasis compared to primary site were common between ER+ and TNBC patients. Anti-apoptotic genes were overexpressed, and microenvironment-regulating genes were downregulated in TNBC metastasis as paired primary sites	[48,49]
20 TNBC patients	57 yrs (40–91)	FFPE	Pathogenic variants in 358 cancer-related genes	Illumina	*MYC* was amplified in 75% of TNBC cases. *TP53*, *AURKA*, and *KDR* were mutated in 30% of patients	[50]
72 NST TNBC patients, 17 ST TNBC patients	49 yrs (22–81)	Plasma	Mutations in 520 cancer-related genes	Illumina	The most frequently mutated genes in NST-TNBC were *TP53* (88.7%), *PIK3CA* (26.8%), and *MYC* (18.3%), and in ST TNBC were *TP53* (68.8%), *PIK3CA* (50%), *JAK3* (18.8%), and *KMT2c* (18.8%)	[57]
96 TNBC patients	55 yrs (36–71)	FFPE	Most frequent mutations in 46 genes with a well-defined role in cancer	Ion Torrent	*TP53, KDR, PIK3CA, ATM, AKT1, KIT, ERBB4, FGFR3,* and *MET* had high mutation frequency in TNBC. *AKT1* rs3730358, *KDR* rs34231037 (c.1444T > 5), *KIT* rs3822214 (c.1621A-C), *TP53* rs28934576 (c.818G > A), and *BRCA1/2* class 5 mutations were associated with poor survival	[58]
85 TNBC patients post-NAC	48 yrs (24–78)	FFPE	Mutations in 182 cancer-related genes	Illumina	In recurrent post- NAC TNBC, there was a high mutation frequency of genes available for targeted therapies: *PTEN* (PIK3CA/AKT pathway inhibitors), *JAK2* (ruxolitinib or tofacitinib), *CDK6, CCDN 1/2/3* (CDK4/6 inhibitors), and *IGF1R* (dalotuzumab)	[59]
56 TNBC patients before-NAC	40 yrs (23–74)	FFPE, plasma	Pathogenic variants in 1977 tumorigenesis-related genes	SOLiD 5500xL	No significant difference was found in the genomic profile of treatment-responsive and resistance TNBCs. *PIK3CA* mutations occur exclusively in *BRCA1* wild-type patients	[61]
7 TNBC patients post NAC with short-DFS (less than 1 yrs); 7 TNBC patients with long-DFS (more than 1 year)	53 yrs (37–71)	FFPE, plasma	Pathogenic variants in 422 cancer-related genes	Illumina	Higher TMB was found in patients with short DFS than in patients with long DFS. Mutations of *PTPN13* and *JARID2* occurred only in the short DFS group	[62]
4647 BC patients (2183 ER/PR+ patients, 237 ER/PR+ and HER2+ patients, 217 HER2+ patients, and 1568 TNBC patients)	NR	FFPE	Mutations in 592 cancer-associated genes	Illumina	18.2% of TNBC had at least one mutation in genes involved in the HR DNA repair pathway. TNBC with HRD had a higher rate of TMB and PDL1 positivity and a higher frequency of alterations in the PIK3CA pathway than patients without HRD	[63]
158 primary and/or metastatic TNBC patients	51 yrs (21–72)	FFPE	Mutations in 468 key cancer-related genes	Illumina	47.4% of TNBC are PD-L1 positive. *CBFB* mutations were more common in PDL1-negative patients than in positive patients. *CBFB* mutations frequently occurred together with *AKT1* and *CDH1* mutations in LAR TNBC	[66]
11 ICPI-treated TNBC patients	50 yrs (NR)	Plasma	Pathogenic alterations in 457 cancer-related genes	Illumina	TNBC patients with *CYP2D6* loss and gain in CNV of *GNAS, BCL2L1, H3F3C, LAG3, FGF23, CCND2, SESN1, SNHG16, MYC, HLA-E,* and *MCL-1* had a shorter PFS after ICPI treatment	[68]
1237 ER+ patients, 1953 HER2+ patients, and 641 TNBC	ER+ patients: 55 yrs (23–89); HER2+ patients: 55 yrs (20–89); TNBC patients: 53 yrs (20–85)	FFPE	TMB, MSI and mutations in 324 cancer-related genes	Illumina	48.6% of ER+, 12.1% of HER2+, and 56.4% of TNBC patients had at least one mutation in ICPI response-associated genes. 2–3% of TNBC patients had mutations in ICPI resistance-associated genes *STK11* and *MDM2*	[78]
305 TNBC patients	49 yrs (NR)	FFPE	Fusion events in NRTK gene	Illumina	IHC and FISH indicated 11.15% of TNBC cases with NRTK fusion events, but NGS validation did not show positivity	[82]

### 3.2. NGS in Ethnic-Specific Molecular Profiling of TNBC

As reported by the American Cancer Society in 2019, the incidence of TNBC is twice as high in women of African American and Hispanic descent than in white women [4]. These incidence rates highlight the genetic population landscape as a potential TNBC risk factor. NGS technologies can to help investigate the role of ethnicity-specific pathogenic alterations in the incidence and progression of TNBC. The related studies are shown in Table 2.

From this perspective, Anwar et al. characterized the copy number variations of 409 cancer genes in 11 Ghanaian metastatic breast cancers (among which 9 were TNBC) using Ion Torrent. Despite the small cohort analyzed, the analysis showed 17 genes with frequent copy number alterations (*SDHC, RECQL4, TFE3, BCL11A, BCL2L1, PDGFRA, DEK, SMUG1, AKT3, SMARCA4, VHL, KLF6, CCNE1, G6PD, FGF3, ABL1*, and *CCND1*), among which, the most common were *RECQL4* (50%) and *SDHC* (60%). A network analysis indicated the involvement of these genes in cell proliferation, apoptosis, and the PIK3CA pathways [83]. Moreover, 13 out of the 17 genes interact with *EZH2*, an epigenetic regulator whose overexpression is associated with metastasis and a negative status of ER and PR in breast tissues [84]. Indeed, another report showed a strong relationship between *EZH2* overexpression and high-grade basal-like breast cancers in a cohort of 169 Ghanaian women [85]. These data provide a basis for further explorations of the pathobiology of breast cancer and TNBC in African and American African women.

The study by Ben Ayed-Guerfali et al. focused on the mutational characterization of *BRCA1/2* in 110 Tunisian women with breast cancer (26 TNBC) and 24 Tunisian women with ovarian cancer. A sequencing analysis was performed using the Illumina platform on plasma samples from the patient cohort. Overall, *BRCA 1/2* mutations occurred in 14.17% of cases and were mainly frameshift (76.9%). The most common genetic alterations were c.1310_1313 delAAGA in the *BRCA2* gene and c.5030_5033 delCTAA in the *BRCA1* gene, which were found in 4% of the breast cancer patients and 20% of the ovarian cancer patients. Interestingly, there was a strong correlation between *BRCA* mutation carriers and TNBC. In fact, 5 of the 26 TNBC patients (19.23%) carried *BRCA* alterations, of which, 4 carried *BRCA1* mutations. This result suggested a higher frequency of BRCA1 carriers than *BRCA2* carriers in the TNBC cases, at least in the Tunisian population [86]. Despite the small number of TNBC cases analyzed, these data are consistent with those reported in other studies [87,88], highlighting the potential of NGS to deepen population-specific molecular TNBC profiles.

Laraqui et al. instead performed a molecular characterization of 30 Moroccan women with early-stage TNBC. Specifically, Illumina-based sequencing was used to investigate the pathogenic variants of 63 cancer-related genes: *AIP, APC, ATM, BAP1, BARD1, BMPR1A, BRCA1, BRCA2, BRIP1, CASR, CDC73, CDH1, CDK4, CDKN2A, CHEK2, EPCAM, FANCM, FH, FLCN, MAX, MCIR, MEN1, MET, MITF, MLH1, MSH2, MSH6, MUTYH, NBN, NF1, NF2, PALB2, PMS2, POLD1, POLE, PTEN, RAD50, RAD51C, RAD51D, RET, SDHA, SDHAF2, SDHB, SDHC, SDHD, SMAD4, SMARCA4, STK11, TMEM127, TP53, VHL, RK1, FAM175, GREM1, MLH3, MRE11, MMSH2, NTHL1, PMS1, RAD51, RAD51B, RINTI, RNF3, RNF43,* and *WRN*. The analysis revealed that 6 of the 30 patients (20%) had known pathogenic variants, the most common of which were associated with *BRCA* (3 patients with *BRCA1* mutations and 2 with *BRCA2* mutations) [89]. As in the previously described Ben Ayed-Guerfali effort, the pathogenic variant c.1310_1313 delAAGA was detected in the *BRCA2* gene in the patients studied, suggesting that this alteration represents a possible founding mutation for TNBC in the North African population. In addition, the analysis revealed 42 variants of unknown/uncertain significance (VUS) in 70% of the patients (21/30), with *ATM* being the gene with the highest frequency of VUS (4/30). This result is to be expected with multigene panel testing and large genes such as *ATM* [90]. Apart from technical limitations, the high number of VUS may indicate a possible new molecular signature of TNBC.

The studies described so far are characterized by a relatively small number of analyzed patients, highlighting the challenges encountered in optimal sample collection from several African environments. Despite this, NGS technologies have achieved promising results in the molecular characterization of TNBC in African women.

**Table 2:** Summary of the experimental methods and main findings of next-generation sequencing (NGS) studies aimed at exploring ethnic-specific molecular signatures in triple-negative breast cancer (TNBC). Abbreviations: BC (breast cancer); CNA (copy number alteration); FFPE (formalin-fixed paraffin-embedded); TNBC (triple-negative breast cancer); and yrs (years).

Patient Population	Median Age (Min-Max)	Source Sample	Target	NGS Platform	Main Finding	Ref.
11 Ghanaian BC patients (9 TNBC patients)	48 yrs (38–64)	FFPE	CNAs in 130 cancer-related genes	Ion Torrent	13 of the 17 genes with CNAs were detected (involved in cell proliferation, apoptosis, and PIK3CA signaling pathways). These genes were associated with overexpression of *EZH2*, an epigenetic regulator frequently altered in Ghanaian TNBCs	[83]
110 Tunisian BC patients (26 TNBC patients)	48 yrs (30–71)	Plasma	Mutations in BRCA1 and BRCA2	Illumina	Mutations c.1310-1313 del AAGA in *BRCA2* and c.5030_5033 in *BRCA1* were found in 4% of BC patients. 5 of 26 TNBC patients had *BRCA* alterations, including 4 with *BRCA1* mutations	[86]
30 early-onset Moroccan TNBC patients	38 yrs (NR)	Plasma	Pathogenic variants in 63 cancer-related genes	Illumina	17% of TNBC cases had *BRCA* alterations. Among them, mutation c.1310_1313 in *BRCA2* was reported. The analysis result showed 42 VUSs	[89]

### 3.3. NGS of Third and Fourth Generation in TNBC Research

As mentioned before, only the Illumina and Ion Torrent platforms have been used for sequencing in the previous chapters. To our knowledge, only few studies have used third- and fourth-generation NGS technologies for a molecular characterization of TNBC, highlighting the need for their optimization for clinical applications. The few TNBC studies in which Pacbio and nanopore sequencing have been used are briefly reported below.

Aganezov et al. performed the whole-genome sequencing of SKBR3, a TNBC cell line [91], using the Illumina platform, Pacbio sequencing, and Oxford Nanopore Technology (ONT) to compare the sequencing efficiency of each method. In addition, the same comparison was performed on organoids from two breast cancer patients obtained from cancer and normal tissues. For all the samples, the analysis showed an excellent accordance of the genetic variants (GVs) detected using the Pacbio and ONT methods, with more than 90% of the GVs being detected by both sequencing techniques. In addition, the long-read methods of Pacbio and ONT detected a large number of GVs missed by the Illumina short-read method. This finding suggests introducing long-read sequencing techniques into routine clinical practice to improve cancer risk assessment, analysis, and treatment. However, the cost of full genomic characterizations using Pacbio and ONT remains significantly higher than that of Illumina sequencing. From the perspective of cost reduction, the study by Aganezov et al. reported the optimal GV detection of long-read methods even at a low average read depth coverage [92]. Unfortunately, this approach may underestimate the heterozygous germline SNPs, which are common in samples from cancer patients [93]. This problem can be circumvented by combining short-read and long-read techniques and continuously reducing the cost of Pacbio nanopore sequencing.

Weirather et al. investigated the possibility of integrating the results of long-read and short-read sequencing methods to allow for a higher precision analysis of fusion genes. This combinatorial method, called IDP-fusion (Isoform Detection and Precision), aims to detect fusion genes, determine fusion sites, and identify fusion isoforms. The flowchart of the IDP-fusion approach begins with the identification of fusion genes using Pacbio wide sequencing. The resulting long reads are aligned to the reference genome to discover the pair fragments that can be mapped to two gene loci. Unfortunately, the boundaries of the selected long fragments cannot be considered as true fusion sites due to the high error rate of Pacbio sequencing. To circumvent this problem, the long fragments are extended by about 2000 base pairs beyond the alignment ends and concatenated to obtain an “artificial reference sequence” (ARS). The ARS thus obtained is then used as a reference to map the high-quality short reads from second-generation sequencing methods, such as Illumina, to identify the true fusion sites. Ultimately, fusion isoform calling involves three steps: the identification of splice sites from long reads, building a candidate isoform library, and estimating the isoform abundance. Weirather et al. tested IDP fusion using a genomic study of MCF-7, a TNBC cell line [94]. Using a panel of 71 common fusion genes in TNBC, the analysis showed a higher fusion gene detection accuracy for the hybrid method (68%) in comparison to the short-read and long-read methods alone (14% and 18%, respectively). In addition, the IDP fusion analysis identified novel fusion genes with tumor interest, such as AIB1-chr1:107073407 (tamoxifen resistance) or TPD52L2-chr17:60952559 (breast cancer proliferation). In summary, long-read and short-read sequencing methods have several inherent limitations, such as a high error rate and miscalling in repetitive genomic regions, respectively [95]. IDP fusion can overcome these problems by combining both techniques, providing a new NGS approach for the molecular characterization of TNBC.

Despite the paucity of efforts to characterize TNBC using third- and fourth-generation sequencing methods, the reported studies have highlighted the possibility of innovative approaches that hold promise for the future use of these technologies in the clinical treatment of TNBC.

## 4. Discussion

Triple-negative breast cancer (TNBC) is a rare and aggressive subtype of breast cancer. The absence of estrogen receptor (ER), progesterone receptor (PR), and human epidermal growth factor receptor 2 (HER2) make current hormone- and HER2-specific therapy inefficient [1]. The high mortality rate of TNBC indicates that the molecular features underlying its tumor development and progression need to be studied in detail. Next-generation sequencing (NGS) methods may be useful for the comprehensive molecular characterization of TNBC in order to discover novel diagnostic, prognostic, and predictive biomarkers. NGS technologies are high-throughput methods that sequence large genomes through massively parallel processes. These methods can be divided into short-read (second generation, such as Illumina, Ion Torrent, and 454 pyrosequencing) and long-read sequencing methods (third and fourth generation, such as Pacbio or Nanopore systems) [30].

NGS techniques have improved our understanding of the human genome and its pathogenic alterations. A total of 3256 TNBC patients have been analyzed using NGS techniques and reported on in this review. The Illumina and Ion Torrent sequencing platforms have revealed a molecular profile characterized by recurrent genetic alterations. Accordingly, *TP53* and *PIK3CA* are the genes with the highest mutation frequency in TNBC patients, followed by other genes involved in cell proliferation, immune response, and DNA repair pathways, such as *MYC, PD-L1, KDR, FGFR3*, and *BRCA1/2*, etc. [47,50,57,58,63]. These results have helped to identify the specific molecular signatures of TNBC subtypes, metastases, or recurrences. For example, the basal-like immunosuppressed (BLIS) and basal-like immune-activated (BLIA) TNBC subtypes are characterized by MYC amplification [50]. In contrast, *AKT1, CDH1, CBFB*, and PD-L1 alterations frequently occur together in the luminal androgenic subtype (LAR) [66]. Additionally, in TNBC without special type (NST), the mutation frequencies in *TP53* and *PIK3CA* are different from those in TNBC with special type (ST), suggesting partially different molecular mechanisms underlying their tumor progressions [57]. NGS has also indicated a *JARID2* mutation (a gene involved in metastasis) and a high tumor burden (TMB) as markers of poor TNBC outcomes [62]. In addition to the diagnostic value of molecular profiling in TNBC, recurrent aberrations may offer new predictive markers and therapeutic targets. The detection of mutations in the immune response genes in TNBC allows for a prediction of the efficiency of treatment with anti-PD-L1 drugs and immune checkpoint inhibitors (ICIP) [66,68,78]. Contrarily, recurrent alterations in the PIK3CA and DNA repair pathways suggest a practical use of PARP inhibitors and drugs targeting the PIK3CA pathway in the treatment of TNBC. Indeed, several clinical trials have confirmed the efficacy of these therapeutic approaches [27,28]. Moreover, NGS techniques may be useful in predicting the efficacy of novel targeted treatments. For example, the high mutation frequency of *AURKA* in TNBC suggests the potential efficacy of AURKA inhibitors such as Alisertib [50]. In contrast, the rare occurrence of NRTK fusion events in TNBC precludes treatment with RTK inhibitors [82].

Comprehensive genomic profiling using NGS is an important tool for studying ethnically specific genetic alterations in TNBC. The latter is characterized by twice the incidence in women of African and Hispanic descent compared to Caucasian women [3,4]. It is well known that, in certain populations, BRCA carrier frequency may be significantly higher due to a founder effect, whereby rare mutations are seen at relatively high frequencies within small, genetically isolated populations. In those populations, founder mutations combine to give a defined carrier risk of developing breast cancer, as this was observed among an Ashkenazi Jewish population, where three common founder mutations, BRCA1_185delAG, BRCA1_5382insC, and BRCA2_6174delT, gave an overall risk of 1 in 40 [96]. In this group, the proportion of hereditary breast cancer is significantly elevated compared to the general population. Studies on Moroccan and Tunisian populations have revealed a high frequency of *BRCA* alterations, especially *BRCA1*. The c.1310_1313 mutation was found in both populations, suggesting that it is a founding mutation for TNBC in women from northeast Africa [83,86]. The sample sizes in the studies from the Moroccan and Tunisian populations were small: overall, founder mutations in populations from resource-poor settings are less well described. Further studies are needed to better understand the implication of the frequency of certain *BRCA* mutations among certain populations, their clinical and prognostic implications, and their potential roles as predictive biomarkers. In contrast, a frequent overexpression of *EZH2*, an epigenetic regulator associated with metastasis and linked to several genes involved in cell proliferation, apoptosis, and the PIK3CA pathway, was found in Ghanaian women. These genes are characterized by a high copy number variation rate in the Ghanaian population [89].

Liquid biopsies refer to different body fluids, such as blood or urine, with different RNA or DNA sources: circulating tumor cells (CTCs), exosomes, and circulating cell-free nucleic acids (cfNAs). These components allow for the real-time monitoring of tumor progression using a non-invasive method [97]. In this review, NGS sequencing was performed on the plasma from a total of 310 TNBC patients in six studies. In our previous review, which focused on circulating cell-free DNA [97], we reported a total of 325 TNBC patients from eight studies that were analyzed using PCR techniques (real-time and digital droplet PCR). This comparison highlights the interest in the use of NGS in liquid biopsy analyses in TNBC. Although NGS has higher sample analysis costs and a lower sensitivity than PCRs, the possibility of obtaining a genetic signature for personalized medicine in TNBC, including therapeutic treatments, is very attractive. Despite the low quantity and high fragmentation of the nucleic acids obtained from liquid biopsies [98,99], they can be sequenced using NGS techniques [100]. In this review, Xiangmei et al. and Lips et al. used plasma DNA as a control to detect germline mutations among those identified using sequencing matched solid biopsies [61,62]. Tan et al. instead examined the copy number variations in the genes in ctDNA, using NGS to predict patient responses to treatment with ICIPs [68]. Li et al., Ben Ayed-Guerfali et al., and Laraqui et al. instead used plasma as their primary tumor DNA source, highlighting the potential of liquid biopsies as a robust and reliable model for genomic studies [57,86,89].

The studies reported in this review were almost all aimed at detecting DNA alterations in patients. Srour et al. [49] instead compared the expression levels of 2567 cancer-related genes in 14 pairs of ER+ primary sites and paired axillary lymph node metastasis (ALN), and in 17 pairs of TNBC primary sites and paired ALN metastasis. These analyses showed that the genes regulating the microenvironment in metastasis were modulated in comparison to the primary sites. Thus, this pilot study demonstrated the potential of NGS techniques for dynamic and comprehensive gene expression profiling. This application may prove useful in clinical research investigating transcriptome changes during tumor progression or treatment responses, for the future clinical management of patients [101].

Third- and fourth-generation sequencing methods were developed to increase the length of the DNA/RNA sequenced using second-generation techniques. Their application still needs to be improved because of their high error rate and high cost. However, their potential is promising, and their steadily decreasing cost and combinations with short-read methods are promising for future clinical applications. For example, Pacbio (third generation) and Oxford Nanopore Technologies (ONT, fourth generation) molecular characterizations of SKBR3 (a TNBC cell line) identified a higher number of pathogenic variations than the second-generation Illumina platform [92]. In addition, a hybrid sequencing method using short and long reads, called IDP fusion, has shown a higher accuracy in detecting gene fusions in MCF -7, another TNBC cell line, than short and long reads alone [95].

Overall, the studies included in this review have some limitations. Only 8 of the 17 studies reviewed had a sample size of more than 100 TNBC patients. One possible explanation for these small sample sizes is the difficulty in recruiting patients without treatment for adequate molecular TNBC profiling. Several of the articles reviewed reported preliminary results based on a small number of studies, which limits the power of their main findings and their application in clinical practice. In addition, several articles contained incomplete or limited clinical information about the patients studied, making it difficult to adequately evaluate the results clinically. A small sample size combined with limited clinical information may underestimate the large heterogeneity of TNBC. The molecular testing using NGS in the articles studied was performed with standard panels of known pathogenic variants in cancer-associated genes. Despite the large size of the panels tested (11 of 17 studies reviewed examined the statuses of at least 300 genes), comprehensive genomic profiling (CGP) may be useful for discovering novel diagnostic or prognostic biomarkers and new therapeutic targets in TNBC. Therefore, further studies are needed to confirm these results.

Ultimately, NGS techniques have deepened the molecular processes regulating the biology of TNBC and led to the development of new therapeutic approaches for the clinical treatment of this tumor. Advances in NGS methods, aimed at faster, less expensive, and more accurate sequencing, are making them increasingly important for TNBC research.

In our opinion, further NGS studies on the immune response and the genes related to DNA repair, as well as therapeutic targets, will bring further advances in the understanding and treatment of TNBC.

## 5. Conclusions

**Figure 4:**
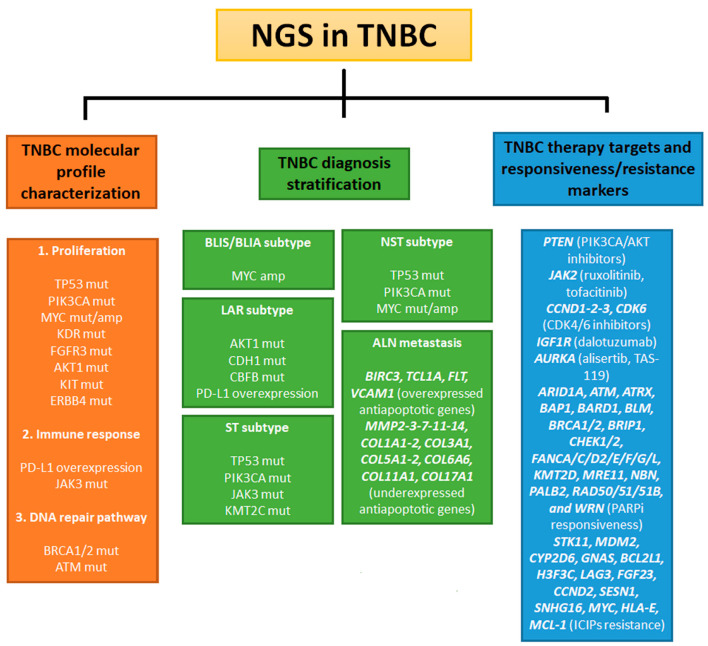
Schematic report of molecular markers suggested by reviewed articles. Abbreviations: mut (mutated); amp (amplified); NGS (next-generation sequencing); TNBC (triple-negative breast cancer); BLIS (basal-like immune suppressed); BLIA (basal-like immune-activated); LAR (luminal androgen receptor); ST (special type); NST (no special type); and ALN (axillary lymph node).

Triple-negative breast cancer (TNBC) shows aggressive clinical behavior, a high risk of recurrence, and poor prognoses, mainly due to its large molecular heterogeneity. Next-generation sequencing (NGS) methods may be useful for comprehensive molecular characterizations of TNBC, in order to discover novel diagnostic, prognostic, and predictive biomarkers and to highlight the ethnically specific genetic alterations in TNBC, with potential therapeutic implications. NGS techniques have revealed recurrent genetic alterations, with *TP53* and *PIK3CA* as the genes with the highest mutation frequencies, followed by other genes involved in cell proliferation, immune response, and DNA repair pathways, such as *MYC, PD -L1, KDR, FGFR3*, and *BRCA1/2* [47,50,57,58,63]. These findings have helped to identify the specific molecular signatures of TNBC subtypes, metastases, or recurrences, which differ according to the different TNBC subtypes, such as luminal androgenic subtype (LAR) [66], TNBC without special type (NST), and TNBC with special type (ST), suggesting partially different molecular mechanisms underlying their tumor progressions [57]. The main findings reported in this review are summarized in Figure 4. In the era of precision medicine, the diagnostic value of molecular profiling in TNBC may provide new predictive markers and therapeutic targets useful for investigating potential personalized therapies for TNBC patients. Most efforts have been directed toward predictive markers of response to therapy or resistance to treatment, indicating the importance of third- and fourth-generation NGS for the molecular profile of TNBC. The major gap to overcome is the entry of third- and fourth-generation NGS into clinical trials for TNBC; this will be achieved by reducing their costs and improving the accuracy of their sequencing results.

## Data Availability

Not applicable.

## List of Abbreviations

ALN	Axillary Lymph Node metastasis
ARS	Artificial Reference Sequence
BLIA	Basal Like Immune Activated
BLIS	Basal Like Immune Suppressed
CAN	Copy Number Alterations
FFPE	Formalin-Fixed Paraffin-Embedded
HRD	Homologous Recombination Deficiency
IBC	Imflammatory Breast Cancer
ICPI	Immune Checkpoint Inhibitors
LAR	Luminal Androgen Receptor
MES	Mesenchymal-like
MGC	Maxam-Gilbert Chemical Cleavage
NAC	Neo Adjuvant Chemotherapy
NGS	Next-Generation Sequencing
NGSH	Next-Generation Sequencing by Hybridization
NGSS	Next-Generation Sequencing by Synthesis
NST	Ductal Carcinoma of Not Special Type
ONT	Oxford Nanopore Technology
pCR	Pathological Complete Response
SBS	Sanger Sequencing By Synthesis
SMRT	Single Molecule Real Time
ST	Ductal Carcinoma of Special Type
TMB	Tumor Mutation Burden
TNBC	Triple-Negative Breast Cancer
VUS	Variations of Unknown/Uncertain Significance
ZMW	Zero-Mode Waveguide
